# Weight-Related Outcomes After Revisional Bariatric Surgery in Patients with Non-response After Sleeve Gastrectomy—a Systematic Review

**DOI:** 10.1007/s11695-023-06630-2

**Published:** 2023-05-20

**Authors:** Stephan Axer, Hans Lederhuber, Franziska Stiede, Eva Szabo, Ingmar Näslund

**Affiliations:** 1 Faculty of Health and Medicine, Örebro University, Campus USÖ, 701 82 Örebro, Sweden; 2 Department of General Surgery, Torsby Hospital, Box 502, 685 29 Torsby, Sweden; 3 Royal Devon University Healthcare NHS Foundation Trust, Church Lane, Exeter, EX2 5DW UK; 4 GP Practice Dr. Fritz Weidinger & Dr. Katharina Klein, Hauptstraße 93, 82327 Tutzing, Germany; 5 Department of Surgery, Faculty of Health and Medicine, Örebro University, Campus USÖ, 701 82 Örebro, Sweden

**Keywords:** Revisional bariatric surgery, Sleeve gastrectomy, Conversion

## Abstract

Weight non-response after sleeve gastrectomy is an emerging issue. This systematic review compared revisional procedures for weight-related outcomes. We searched several databases for relevant articles and included adult patients with revisional bariatric procedures after primary sleeve gastrectomy. Twelve trials with 1046 patients were included, covering five revisional procedures. There were no randomised controlled trials, and 10 studies had a critical risk of bias. Significant variations in inclusion criteria, therapy benchmarks, follow-up schemes, and outcome measurements were observed, preventing meaningful comparison of results. Evidence-based treatment strategies for weight non-response after sleeve gastrectomy cannot be deduced from the current literature. Prospective studies with well-defined indications, standardised techniques, and strict adherence to outcome measurements are needed.

**Supplementary Information:**

The online version contains supplementary material available at 10.1007/s11695-023-06630-2.

## Introduction

The acknowledgement of obesity as a chronic disease in 2013 by the American Medical Association (AMA) was a key milestone in history of bariatric surgery [1]. Revisional bariatric surgery as a second-line treatment in patients with primary or secondary non-response in terms of weight loss became increasingly shaped by the comprehension of the chronic character of obesity. It is of importance to evaluate the treatment results in detail, to identify an eventual technical failure of the primary procedure, and to consider second-line treatment.

Sleeve gastrectomy (SG) has gained popularity during the last decade and relegated gastric bypass (GBP) from its status as the most frequently performed procedure for treatment of obesity and related diseases [2]. The reasons for this development are diverse; SG is considered technically more straightforward, patients explicitly request this type of procedure, and it offers the opportunity to escalate the therapeutic effects by proceeding with a plus-operation [3].

With increasing numbers of procedures performed globally, reports about drawbacks of SG became more frequent. Reflux, oesophagitis, and development of Barrett mucosa are common indications for revisional surgery. Conversion from SG to GBP is a current surgical approach with seemingly robust scientific footing [4]. Even other procedure-related complications, such as stricture or twisting of the sleeve, chronic fistulation, and intra-thoracic herniation, are rectifiable by GBP [5]. This procedure appears to be the omnipotent remedy when it comes to resolution of sleeve-related issues [4]. Hence, it is not surprising that GBP is even regarded to be the preferable approach to treat primary or secondary non-response as well as relapse or persistence of obesity-related comorbidity after previous SG [6]. In addition, alternative approaches have recently been raising attention: re-sleeve gastrectomy (re-SG) [7], conversion to one anastomosis gastric bypass (OAGB) [8], duodenal switch with biliopancreatic diversion (DS) [9], or single anastomosis duodeno-ileal bypass (SADI) [10]. So far, there is a lack of evidence to support decision-making and the surgical approach is often rather based on local or individual protocols than on evidence-based guidelines.

This systematic review of the literature aimed at assessing and comparing postoperative weight-related outcomes after various bariatric surgical revisional procedures after primary sleeve gastrectomy. Based on these data, we attempted to propose a treatment strategy and identify areas for future research in this field.

## Methods

### Study Design and Protocol Registration

We performed a systematic review of randomised and non-randomised controlled trials on the postoperative outcomes of revisional surgery for primary and secondary non-response after sleeve gastrectomy. The Cochrane Handbook for Systematic Reviews of Interventions (version 6.2) [11] was used to design the study protocol. We reported this review in accordance with the updated Preferred Reporting Items for Systematic Review and Metaanalysis guidelines (PRISMA 2020) and checklists and extensions (PRISMA 2020, PRISMA-Abstracts 2020, PRISMA-S) [12]. PRISMA checklists are provided in Supplementary Tables 1–3. Prior to starting the review, we established the review methodology and registered the systematic review 26/03/2021 in the international prospective register of systematic reviews PROSPERO (CRD42021236342, available at https://www.crd.york.ac.uk/PROSPERO/display_record.php?RecordID=236342). Amendments to the original protocol became necessary prior to title and abstract screening. Details about the reason for amendments can be found in the online PROSPERO protocol.

### Eligibility Criteria and Search Strategy

We created a research question, using the “Population, Intervention, Comparison, Outcome and Search Strategy” (PICOS) framework. Asking the question “What are the postoperative outcomes after reversing SG to other bariatric procedures due to inadequate weight loss or weight recurrence?”, we established the following PICOS, guiding the eligibility of studies into the systematic review:

- Population (P): adult patients with open or laparoscopic SG that has been revised to any other bariatric procedure (open or laparoscopic)
- Intervention (I): revision of SG to any other bariatric procedure
- Comparison (C): any revisional bariatric procedure not identical to the intervention
- Outcome (O): weight loss after revised bariatric surgery
- Study design (S): randomised controlled and non-randomised controlled trials

Based on this PICOS, we used the single platform HDAS (Healthcare Database Advanced Search) to perform a comprehensive search for articles published between 1/1/2000 and 30/4/2021 in the databases PubMed/MEDLINE, Cochrane Library database, BNI, CINAHL, EMBASE, EMCARE, and ClinicalTrials.gov. The language of published articles was restricted to English. No published search filters were used. We screened reference lists of included articles for further eligible articles but did not perform a formal search for grey literature (neither grey literature databases nor contacting authors). The search was last run and updated 31/3/2022.

Two reviewers (SA and HL) independently constructed a search term and condensed it into one final search term that provided the most comprehensive result lists: ((revision OR revisional OR conversion) AND (“sleeve” OR “sleeve gastrectomy” OR “SG”) AND (“weight regain” OR “weight loss failure” OR “weight recidivism” OR “inadequate weight loss” OR “insufficient weight loss”)).

Exclusion criteria for this systematic review were:

- age of the study population < 18 years
- the performed revisional bariatric procedure was an endoscopic sleeve gastroplasty (ESG)
- the indication for revisional surgery was persistence, relapse, or devo-no occurrence of obesity-related only
- the article was a technical report, video vignette, systematic review, editorial, guideline, single case report, case series, cohort study, non-comparative observational study, or a comparative study with less than 20 included individuals.

The retrieved articles were managed using the bibliographic software Papers^3^ for Mac (Version 3.4.25, Digital Science & Research Solutions, Inc.).

### Interventions and Outcomes

All revisional bariatric procedures after a SG procedure were assessed and described in detail.

Primary outcomes included weight change after revisional surgery, measured as body mass index (BMI), percental total body weight loss (%TWL), percental excess weight loss (%EWL), or percental excess BMI loss (%EBMI loss). No secondary outcomes were included.

### Study Selection and Data Extraction

Articles were run through automatic deduplication, using Rayyan SaaS web application [13]. Two reviewers (SA and FS) then independently analysed titles and abstracts of all articles resulting from the comprehensive database search, using Rayyan. They grouped articles into “include”, “exclude,” and “possibly include”. After all articles had been screened, disagreement was either solved by discussion and re-evaluation or, if no consensus could be achieved, a third reviewer (HL) made the final decision after reviewing the article in question. Two authors (SA and FS) then independently performed a full-text assessment of the remaining articles on the basis of the aforementioned eligibility criteria. Again, if no consensus could be reached, a decision was reached either by discussion or by decision of a third reviewer (HL).

Two reviewers (SA and FS) extracted data from the included studies and merged them into one dataset.

### Risk of Bias Assessment

The study selection rendered no randomised controlled clinical trial (RCT) eligible for inclusion in the presented systematic review. Details on a priori planned quality assessment for RTCs are hence omitted. Two authors (HL and SA) assessed the methodological quality of the non-randomised studies, using the risk of bias in non-randomised studies of interventions tool (ROBINS-I). They applied the ROBINS-I-tool as described previously [14]. In short, the tool assesses seven domains (2 pre-intervention, 1 intervention, and 4 post-intervention) of potential bias and provides one global domain on the overall risk of bias. The individual domains and global risk of bias are classified as:

- “Low risk of bias: the study is comparable to a well-performed randomised trial with regard to this domain
- Moderate risk of bias: the study is sound for a non-randomised study with regard to this domain but cannot be considered comparable to a well-performed randomised trial
- Serious risk of bias: the study has some important problems in this domain
- Critical risk of bias: the study is too problematic in this domain to provide any useful evidence on the effects of intervention
- No information: there is not enough information on which to base a judgement about risk of bias for this domain [14]

As per ROBINS-I tool guidance, studies that were assessed as at critical risk of bias in at least one domain were rated as at critical risk of bias in the global domain. These studies were as per ROBINS-I protocol not included into the discussion and conclusions of this review, as they were deemed too problematic to provide any useful evidence.

Inter-rater agreement was calculated using QuickCalc Cohen’s Kappa (K) calculator (GraphPad, San Diego, USA. Available at https://www.graphpad.com/quickcalcs/kappa1/).

### Synthesis of the Data

After preliminary screening of the studies, it became obvious that the studies differed widely in methodology and reported outcomes. We decided that a meta-analysis would be inappropriate, and a systematic review was performed instead.

## Results

### Study Selection

**Fig. 1:**
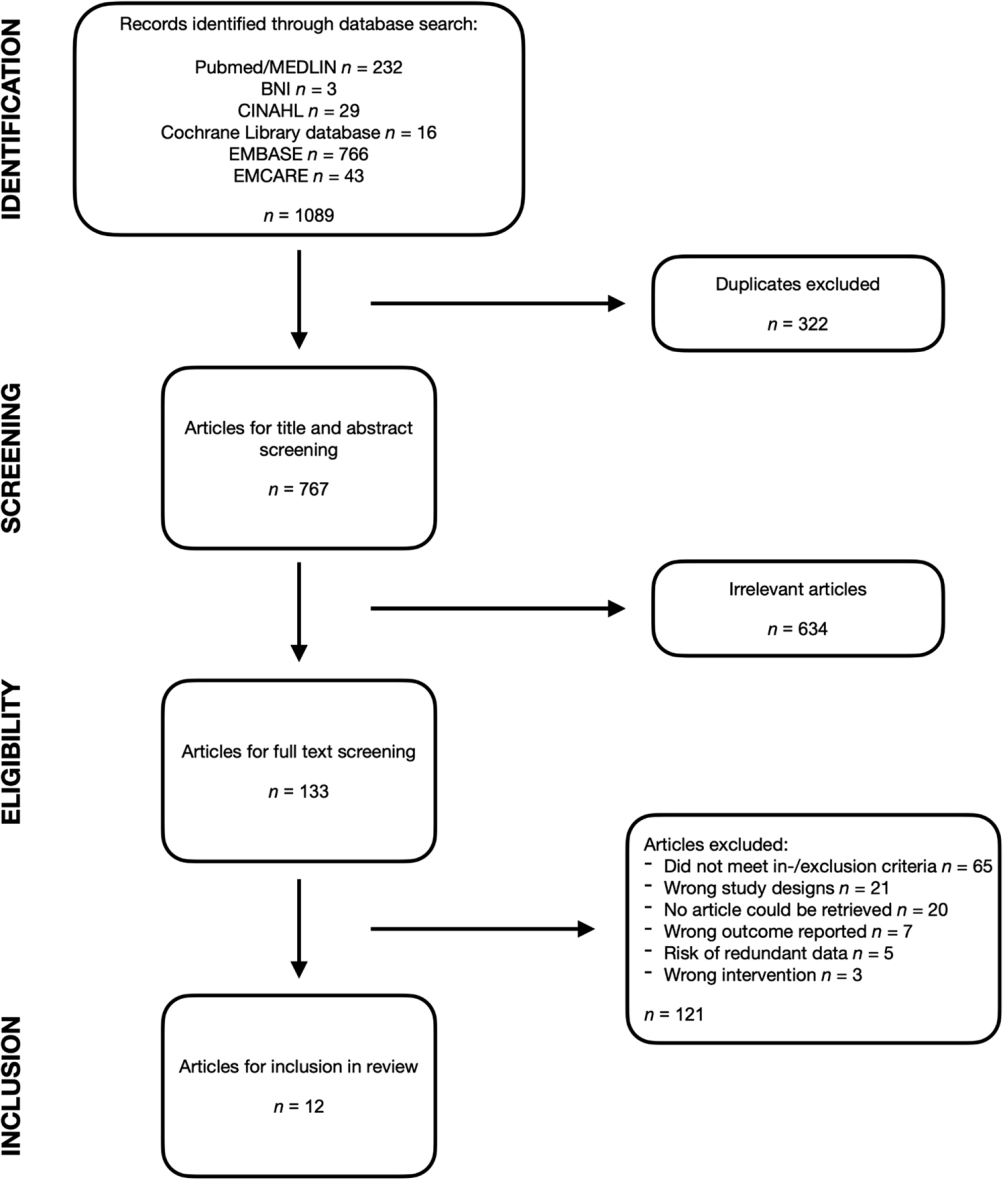
PRISMA flow chart

We retrieved a total of 1089 citations from the databases’ research (232 from PubMed/MEDLINE, 3 from BNI, 29 from CINAHL, 16 from Cochrane Library database, 766 from EMBASE, 43 from EMCARE). After removal of duplicates, we screened the title and abstract of 767 articles, after which a total of 133 articles remained for full-text screening. Figure 1 shows the PRISMA flow chart that concluded with 12 studies meeting the criteria for data extraction. Inter-rater agreement for full-text screening showed a Cohen’s *K* = 0.744 (95% CI 0.549–0.940), indicating substantial agreement. Studies that were excluded during full-text assessment are listed in Supplementary Table 4 and Fig. 1, together with a short explanation for exclusion.

### Study Characteristics

**Fig. 2:**
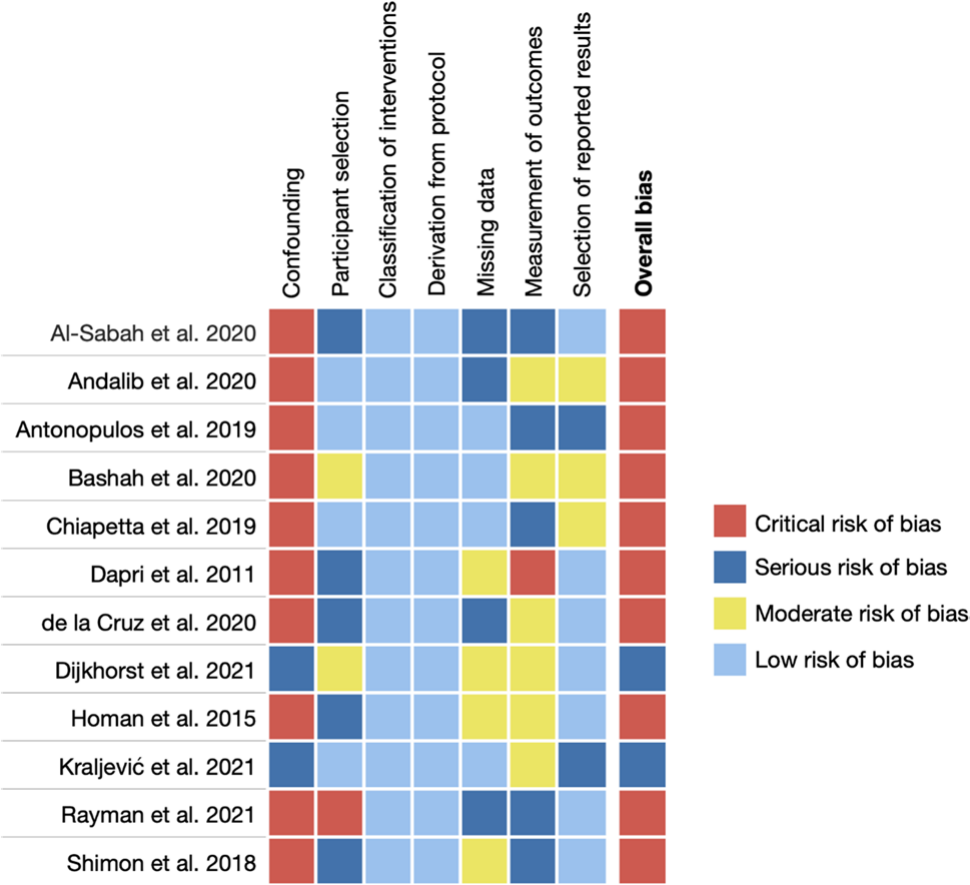
Risk of bias assessment according to ROBINS-I tool for non-randomised studies. The shown judgement categories are low risk of bias (the study is comparable to a well-performed randomised trial with regard to this domain); moderate risk of bias (the study is sound for a non-randomised study with regard to this domain but cannot be considered comparable to a well-performed randomised trial); serious risk of bias (the study has some important problems in this domain); critical risk of bias (the study is too problematic in this domain to provide any useful evidence on the effects of intervention)

**Fig. 3:**
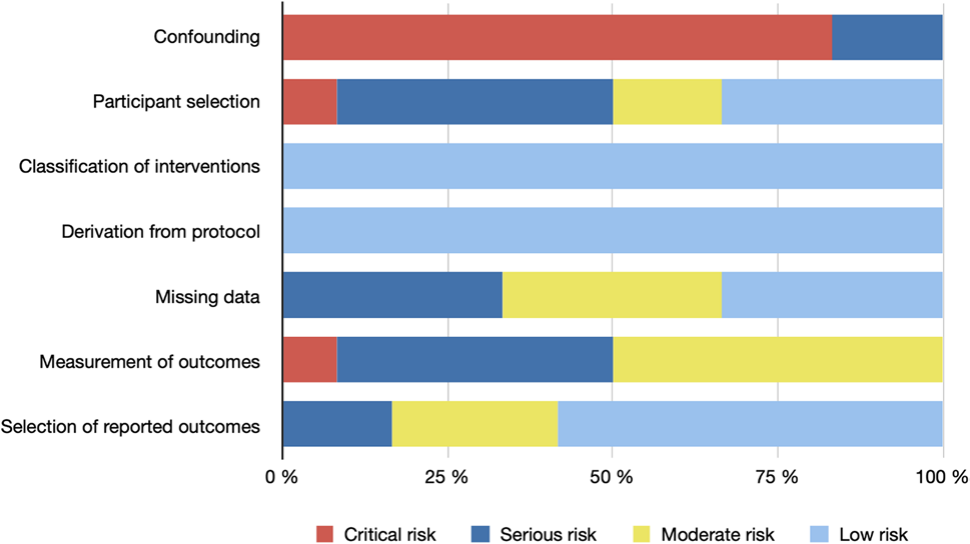
Summary of ROBINS-I assessment per domain across studies

None of the 12 eligible studies could be assessed as at overall low risk of bias. Using the ROBINS-I tool as described above, we assessed two studies at serious risk [15, 16] and 10 studies as at critical risk of bias [9, 17–25]. Inter-rater agreement had a Cohen’s *K* = 0.714 (95% CI 0.334–1.000), indicating substantial agreement (one disagreement in 12). The detailed assessment of risk of bias is shown in Figs. 2 and 3.

Included studies and their characteristics are shown in Table 1. Apart from three multi-centre studies [16, 19, 24], publications were based on the retrospective analysis of prospectively collected databases at single institutions. None of the studies was a randomised controlled trial. The median time of inclusion was 7 years (range 1.5–11.0 years). Ten studies were published within 2 years after completion of inclusion.

**Table 1:** Summary of study characteristics

Author	Country	Time of inclusion	Year of publication	Journal	Revisional procedure	Ethics
Al Sabah et al. [17]	Kuwait	2008–2019	2020	SOARD	GBP/Re-SG	Y
Andalib et al. [18]	Canada	2010–2018	2020	Surg Endo	Re-SG/GBP/DS/SADI	Y
Antonopoulos et al.[19]	France	2010–2017	2019	Obes Surg	GBP/Re-SG	Y
Bashah et al. [20]	Qatar	2016–2017	2020	Obes Surg	SADI/OAGB	Y
Chiappetta et al. [21]	Germany	2014–2016	2019	Obes Surg	OAGB/GBP	Y
Dapri et al. [22]	Belgium	2003–2009	2010	SOARD	DS/Re-SG	N
De la Cruz et al. [23]	Germany	2013–2018	2020	SOARD	SADI/OAGB	Y
Dijkhorst et al. [16]	Netherlands	2007–2017	2021	Obes Surg	SADI/GBP	Y
Homan et al. [9]	Netherlands	2008–2013	2015	SOARD	DS/GBP	Y
Kraljević et al. [15]	Switzerland	2012–2016	2021	Obes Surg	OAGB/GBP	Y
Rayman et al. [24]	Israel	2013–2019	2021	Obes Surg	OAGB/GBP	Y
Shimon et al. [25]	Israel	2006–2016	2018	Obes Surg	DS/GBP	Y

*GBP*, gastric bypass; *Re-SG*, re-sleeve gastrectomy; *DS*, duodenal switch; *SADI*, single anastomosis duodeno-ileal bypass; *OAGB*, one anastomosis gastric bypass

Nine studies evaluated the effects of GBP as a revisional procedure [9, 15–19, 21, 24, 25]. A total of 405 patients were included in these studies. OAGB was assessed in five studies [15, 20, 21, 23, 24] which comprised 281 patients. Andalib et al. [18], Dapri et al. [22], Homan et al. [9], and Shimon et al. [25] published data on their results when performing DS as a revisional procedure after SG in 87 patients. Four studies evaluated SADI as revisional procedure in 154 patients [16, 18, 20, 23]. A total of 119 patients in four studies underwent re-SG [17–19, 22].

### Patient Characteristics

Mean age ranged from 37 [17] to 50 years [9], and the proportion of female participants between 42% [22] and 92% [18]. Female gender was predominant in most studies. A history of previous bariatric surgery before SG was reported by Antonopoulos et al. and Al-Sabah et al. [17, 19].

### Definition of Inclusion Criteria

Clear inclusion criteria for revisional bariatric surgery due to weight issues were absent in two studies [17, 22]. Homan et al. [9] and Dijkhorst et al. [16] applied the IFSO guidelines [26] for bariatric surgery as a criterion for revisions. Excess weight loss of less than 50% was the most frequently quoted indication [9, 15, 18, 19, 21, 24]. Definition of weight regain was inconsistent, e.g. “… ≥ 20% weight regain of the weight lost …” [18] or “…WR was defined as achieving an initial 50% EWL but eventually regaining weight…” [24]. Detailed descriptions of inclusion criteria from all included studies are provided in Supplementary Table 5.

### Definition of Outcomes

In one study, weight loss outcomes were not pre-defined [25]. In six studies, %EWL and %TWL were used to describe weight loss [9, 15, 18, 20, 21]. Percental total body weight loss (%TWL) was the single definition of outcome in three publications [16, 23, 24], and percental excess weight loss (%EWL) in another three studies [17, 19, 22]. Only four studies described whether they calculated weight differences based on weight/BMI at the time of index SG or at time of revision. [16, 20, 22, 24]. Verbatim definitions of outcomes are listed in Supplementary Table 6.

### Follow-up

A pre-defined follow-up schedule was not described in five studies [9, 18, 22, 24, 25]. The widest range of time to last follow-up (7–78 months) was reported by Rayman et al. In this publication, the mean follow-up time differed between the two study groups: 25.5 months after OAGB vs. 35.0 months after GBP [24].

A total of 1046 patients were included in 12 studies. The cumulative follow-up rates re-calculated as the ratio between available and included patients were 58% (*n* = 602) after 1 year, 34% (*n* = 353) after 2 years, 28% (*n* = 291) after 3 years, 8% (*n* = 80) after 4 years, and 4% (*n* = 43) after 5 years. Follow-up rates grouped by revisional procedure are listed in Supplementary Table 7.

### Study Results by Type of Revisional Procedure

Table 2 display number of patients included per study, as well as mean BMI (kg/m^2^) at the time of SG, at the time of revision, and at the last follow-up. Technical details of the revisional procedure are summarised, and time intervals between primary and secondary surgery are given in months. Data at follow-up include date (fix or mean), percentage completeness of follow-up based on the ratio between available and included patients, BMI, %TWL, %EWL, or %EBMI loss.

**Table 2:**
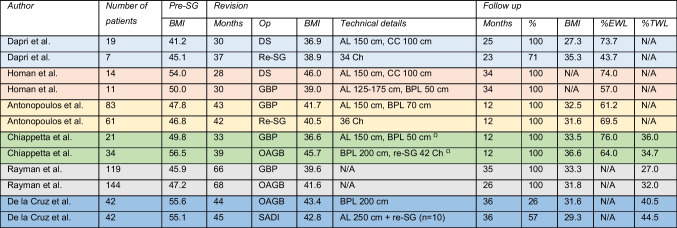
Results calculated on basis of BMI/weight at time of SG

*SG*, sleeve gastrectomy; *BMI*, body mass index (kg/m^2^); *%EWL*, percental excess weight loss; *%TWL*, percental total weight loss; *DS*, duodenal switch; *AL*, alimentary limb; *CC*, common channel; *Re-SG*, re-sleeve gastrectomy; *GBP*, gastric bypass; *BPL*, biliopancreatic limb; *OAGB*, one anastomosis gastric bypass; *SADI*, single anastomosis duodeno-ileal bypass

Supplementary Table 8 describes the technical details of SG and the different revisional procedures.

## Discussion

The aim of this systematic review was to compare postoperative weight-related outcomes after different revisional bariatric procedures for primary or secondary weight non-response after primary SG. Twelve non-randomised trials qualified for inclusion. Risk of bias was critical in 10 studies, leaving only two studies that qualified for in-depth review of the results. From these, the following conclusions could be drawn: (1) OAGB with a 200-cm biliopancreatic limb (BPL) results in as much additional %EWL as GBP with a BPL of 100–150 cm and a common channel of 100 cm at 3 years follow-up [15]. (2) Weight loss after conversion from SG to SADI is significantly higher compared to conversion from SG to GBP at the time of 4 years follow-up [16].

While SG is still gaining popularity among patients and surgeons, the awareness is growing that this procedure is afflicted with primary or secondary weight non-response, commonly termed as *weight loss failure*, *insufficient weight loss*, or *weight regain*. Surgical strategies to meet weight non-response are manyfold, but despite a plethora of trials exploring weight outcome after revisional bariatric surgery, statements and recommendations regarding revisional bariatric surgery as a second-line treatment after SG are contradictory [6, 27] and not systematic. The studies not included into the synthesis of results in this review actually cast more light on current problems in research on revisional bariatric surgery than the studies that were included: (1) not a single randomised controlled trial could be identified. (2) Most trials were non-comparative or retrospective in nature. (3) All trials included into this review had a problem with high risk of bias, mainly due to a combination of the nature of the respective trial and additional methodological shortcomings. (4) The description of the relation between eligible and included study participants was insufficient. As a result, it is unclear whether the patients included in the studies are truly representative of the broader population or just the tip of the iceberg.

It has previously been shown that confounding is the most common reason to rate a non-randomised trial as being at critical risk of bias [28]. This is in line with our assessment where 10 studies were confounded by age, gender, weight/BMI at the time of SG, time interval between the two procedures surgery, choice of revisional procedure, and weight/BMI at the time of revision. Only Dijkhorst et al. identified several relevant confounding factors and adjusted weight loss results by sensitivity analyses [16]. Kraljević et al. did not adjust for confounding factors but described a decision algorithm for which patients received which procedure [15].

While bias due to confounding is a common issue in non-randomised controlled trials, a variety of methodological issues in these 12 studies need highlighting such as study design, technical aspects of the revisional procedure, timeframe of inclusion, follow-up, and outcome reporting.

Nine studies were purely designed for revisional surgery due to weight non-response. When other indications for conversion or correction such as functional issues or complications are included, smaller subgroups must be analysed [15, 16, 21]. Furthermore, when coexisting indications determine the type of revisional procedure, weight and BMI effects can hardly be compared [21].

Another issue of concern in this systematic review was technical aspects. The history of bariatric surgery has been characterised by innovations, technical progress, and the implementation of new techniques The larger the timeframe for inclusion of patients becomes, the greater is the probability that procedural modifications are likely to be adopted within that frame [16]. The use of modified procedures within the same patient group compromises internal validity and should be avoided [20, 25].

Primary and secondary weight non-response are inconsistently defined in the literature [29–31]. Hence, thresholds for an escalation of therapy by revisional surgery are at the surgeon’s discretion. None of the quoted definitions of primary or secondary non-response in any publication coincided with another. In other words, the inclusion criteria varied considerably. As long as bariatric surgeons disagree on thresholds for revisional bariatric surgery or any other type of second-line treatment, the comparability of research in this field will be limited.

**Fig. 4:**
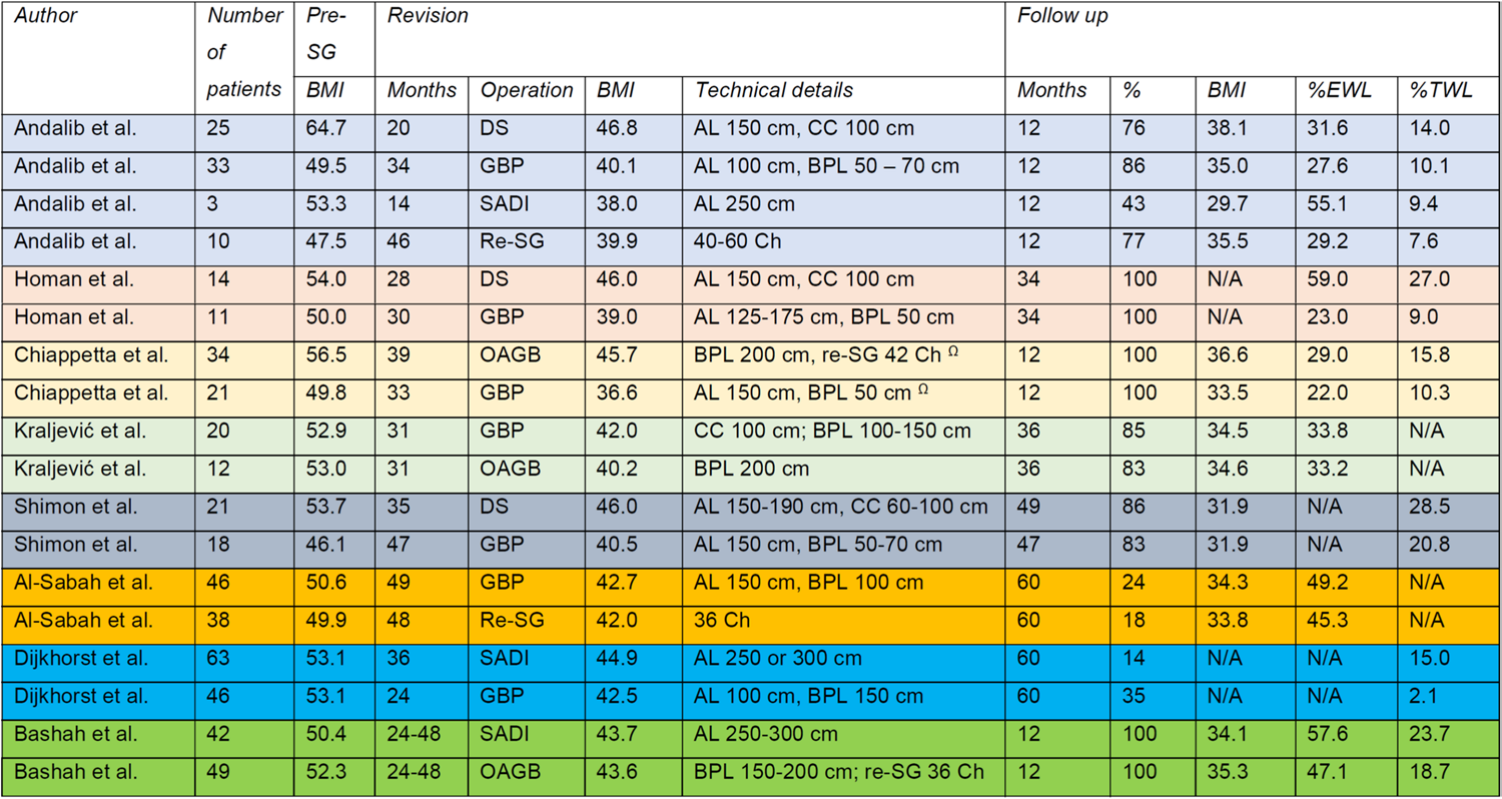
Results calculated on basis of weight/BMI at time of revision

Guidelines on how to report results after revisional bariatric surgery are lacking [32]. According to Brethauer et al., reports on weight loss after bariatric surgery should include four different parameters: the initial BMI of the cohort, change in BMI, %TWL, and %BMI loss or %EWL. The meaningfulness of the different outcomes and their contribution to the interpretation of results can be questioned. The pros and cons of percental excess weight loss versus total body weight loss have been discussed previously [33]. None of the 12 publications provided a complete set of outcome measurements. Furthermore, the comparability of treatment results was impaired by the application of different reference points. Weight and BMI results were either calculated based on data collected at the time of SG [19, 22–24] or at the time of revisional surgery (Fig. 4) [15–18, 20, 25]. To choose the index operation as reference point implies a higher interest in the effects of bariatric surgery as an entity offering the opportunity to analyse individual weight trajectories [34]. When weight and BMI results are calculated with regard to data at the time of revision, the effectiveness of the conversional or corrective procedure is in the main focus of interest. This type of report is preferable when data on the index operation is missing. Regardless which reference point is applied, a clear definition must be provided [16, 20, 22, 24].

Strong recommendations for follow-up have previously been made [33]. In an ideal setting, study results should be based on comprehensive information [35]. The completeness of follow-up determines the validity of a study [36]. The plausibility of follow-up rates and the trustworthiness of a study are increased by reporting the total number of individuals included, and the patient availability and eligibility at each point of follow-up.

This systematic review has some limitations. The small number of low bias trials can indeed impact the generalizability of the conclusions drawn from the review. By limiting study eligibility to comparative trials, we excluded a number of articles that retrospectively assessed weight results. We would argue however that inclusion of studies with a single intervention and no comparator would not have provided a better insight. Another limitation is for sure the exclusion of the few publications that were from the same author or research group. While this reduces the risk of redundant inclusion of patients on the one hand side, it implicates the risk of falsely low early follow-up rates on the other hand side. We think that short-term results after any bariatric surgery are mostly of interest in terms of complications. With stable weight usually reached after 18–24 months postoperative, we considered the risk of over-reporting of short-term results as higher.

Evidence-based treatment strategies for patients with weight non-response after sleeve gastrectomy can hardly be deduced from the current literature. SADI appears to be superior compared to GBP inducing more weight loss up to 5 years after revisional surgery. GBP with a long BPL and a short common channel seems to be as effective as OAGB with 200 cm BPL regarding additional %EWL after 3 years. But the bottom line remains that any future trial investigating into the outcomes of revisional bariatric surgery must apply distinct pre-defined benchmarks, prospective study protocols with scheduled follow-up, uniform surgical technique, and strict abidance by recommended outcome measurements.

## Supplementary Information

Below is the link to the electronic supplementary material.

Supplementary Fig. 1 Reasons for study exclusion. The reasons are displayed as number of studies per category. (PNG 169 kb)

High resolution image (TIF 658 kb)

Supplementary Table 1 (DOCX 25 kb)

Supplementary Table 2 (DOCX 19 kb)

Supplementary Table 3 (DOCX 31 kb)

Supplementary Table 4 (DOCX 24 kb)

Supplementary Table 5 (DOCX 14 kb)

Supplementary Table 6 (DOCX 14 kb)

Supplementary Table 7 (DOCX 14 kb)

Supplementary Table 8 (DOCX 46 kb)

## Data Availability

The authors declare that all relevant data used to conduct this review are included in the manuscript and the accompanying supplements.
