# Supplementary figures and images for: Weight-Related Outcomes After Revisional Bariatric Surgery in Patients with Non-response After Sleeve Gastrectomy—a Systematic Review
Source: Obes Surg. 2023 May 20;33(7):2210–8. doi: 10.1007/s11695-023-06630-2 (PMC10289909; doi:10.1007/s11695-023-06630-2)

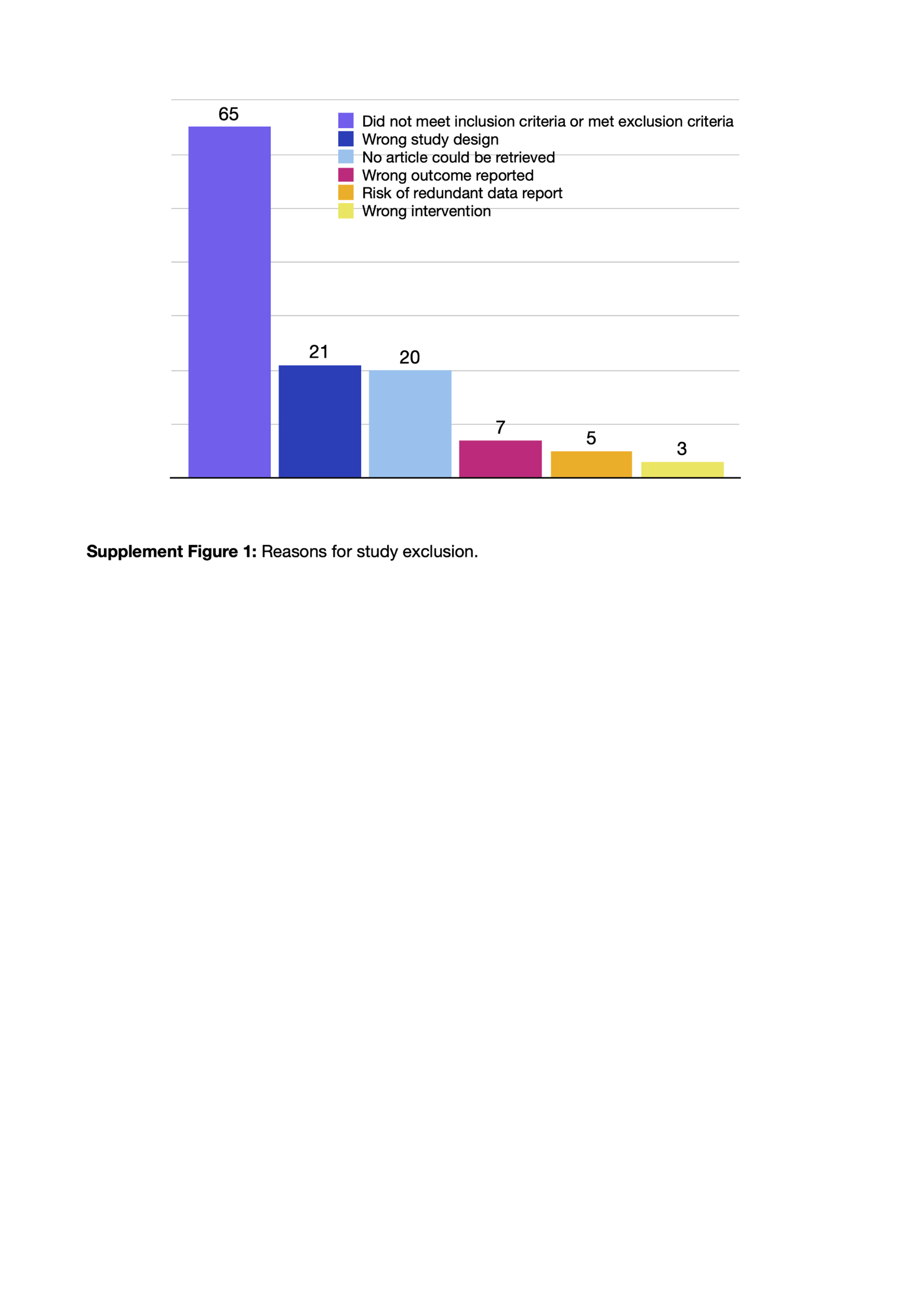

Supplement: Supplementary file 1 — Reasons for study exclusion. The reasons are displayed as number of studies per category. (PNG 169 kb) [file 11695_2023_6630_Fig5_ESM.png]

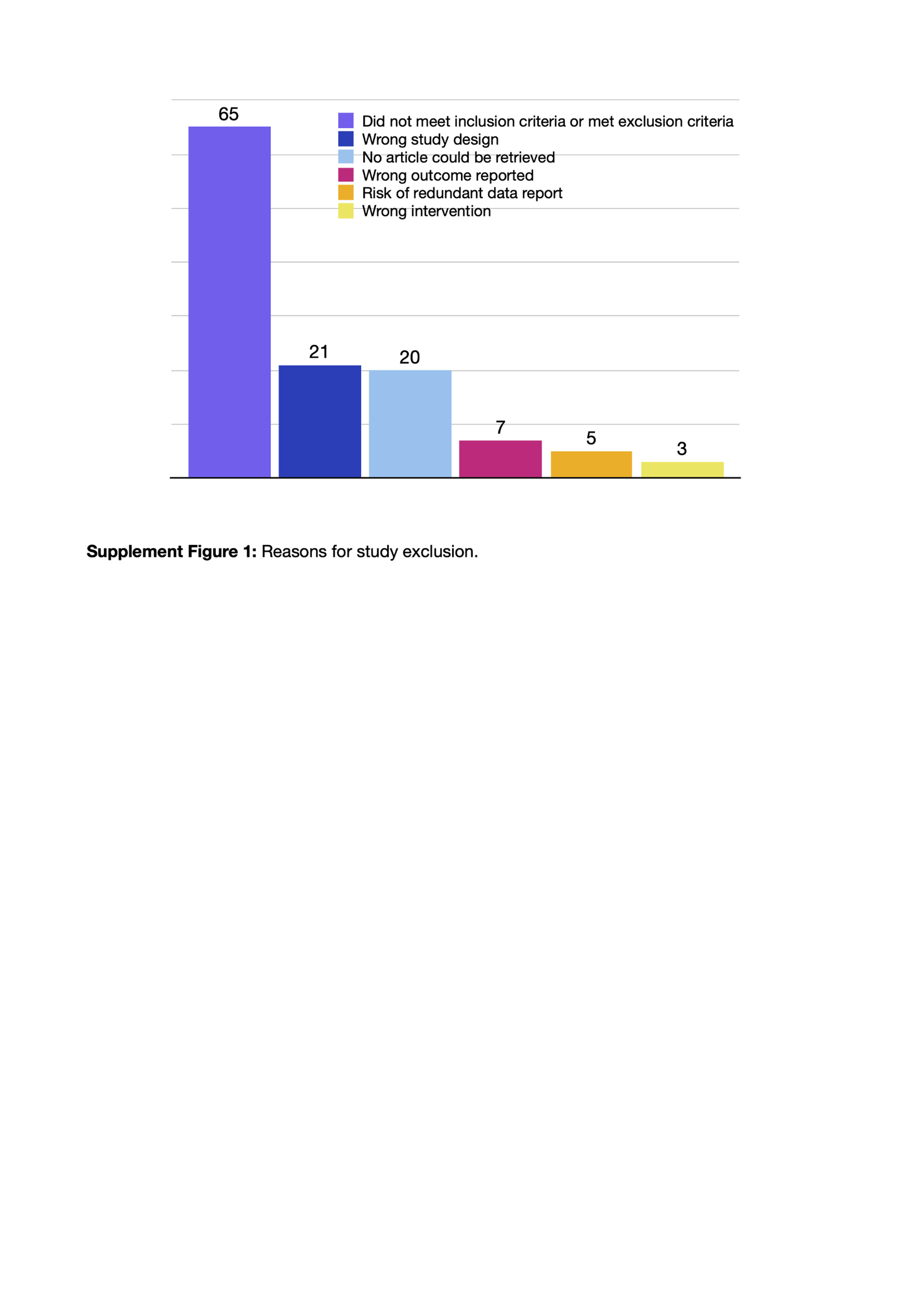

Supplement: Supplementary file 2 — High resolution image (TIF 658 kb) [file 11695_2023_6630_MOESM1_ESM.tif]
