# Supplementary material for: Weight-Related Outcomes After Revisional Bariatric Surgery in Patients with Non-response After Sleeve Gastrectomy—a Systematic Review
Source: Obes Surg. 2023 May 20;33(7):2210–8. doi: 10.1007/s11695-023-06630-2 (PMC10289909; doi:10.1007/s11695-023-06630-2)
Supplement: Supplementary file 6 — (DOCX 24 kb) [file 11695_2023_6630_MOESM5_ESM.docx]

**Supplementary Table 4:** List of articles excluded during full-text screening and reason for exclusion.

| Reference | | Reason for exclusion |
| --- | --- | --- |
| Abdelgawad et al. | 2016 | Did not meet inclusion criteria or met exclusion criteria |
| Ahmad Salem et al. | 2019 | Did not meet inclusion criteria or met exclusion criteria |
| Ahmad et al. | 2017 | Did not meet inclusion criteria or met exclusion criteria |
| Aleassa et al. | 2018 | Did not meet inclusion criteria or met exclusion criteria |
| Aleassa et al. | 2018 | Did not meet inclusion criteria or met exclusion criteria |
| Al Sabah et al. | 2016 | Did not meet inclusion criteria or met exclusion criteria |
| Al Sabah et al. | 2017 | No article could be retrieved |
| Al Sabah et al. | 2018-1 | Did not meet inclusion criteria or met exclusion criteria |
| Al Sabah et al. | 2018-2 | Risk of redundant data report |
| Al Sabah et al. | 2019 | Did not meet inclusion criteria or met exclusion criteria |
| Alsharqawi et al. | 2015 | Did not meet inclusion criteria or met exclusion criteria |
| Alshurafa et al. | 2017 | No article could be retrieved |
| Alshurafa et al. | 2019 | Did not meet inclusion criteria or met exclusion criteria |
| Aly et al. | 2017 | Did not meet inclusion criteria or met exclusion criteria |
| Balibrea et al. | 2017 | No article could be retrieved |
| Barajas-Gamboa et al. | 2019 | Did not meet inclusion criteria or met exclusion criteria |
| Bereczky et al. | 2017 | No article could be retrieved |
| Bereczky et al. | 2018 | Did not meet inclusion criteria or met exclusion criteria |
| Bhandari et al. | 2017 | Did not meet inclusion criteria or met exclusion criteria |
| Bhandari et al. | 2019-1 | Did not meet inclusion criteria or met exclusion criteria |
| Bhandari et al. | 2019-2 | No article could be retrieved |
| Bhandari et al. | 2019-3 | Wrong study design |
| Biertho et al. | 2014 | Did not meet inclusion criteria or met exclusion criteria |
| Biertho et al. | 2018 | Wrong intervention |
| Boru et al. | 2018 | Did not meet inclusion criteria or met exclusion criteria |
| Boudreau et al. | 2019-1 | Did not meet inclusion criteria or met exclusion criteria |
| Boudreau et al. | 2019-2 | Did not meet inclusion criteria or met exclusion criteria |
| Boudreau et al. | 2019-3 | Did not meet inclusion criteria or met exclusion criteria |
| Boza et al. | 2015 | Did not meet inclusion criteria or met exclusion criteria |
| Boza Wilson et al. | 2019 | No article could be retrieved |
| Carmeli et al. | 2014 | Did not meet inclusion criteria or met exclusion criteria |
| Carmeli et al. | 2015 | Did not meet inclusion criteria or met exclusion criteria |
| Casillas et al. | 2016 | Wrong study design |
| Chang et al. | 2018 | Did not meet inclusion criteria or met exclusion criteria |
| Chouillard et al. | 2019-1 | Did not meet inclusion criteria or met exclusion criteria |
| Chouillard et al. | 2019-2 | Did not meet inclusion criteria or met exclusion criteria |
| Coleman et al. | 2015 | Did not meet inclusion criteria or met exclusion criteria |
| Contreras et al. | 2019 | Did not meet inclusion criteria or met exclusion criteria |
| Coste et al. | 2021 | No article could be retrieved |
| De Angelis et al. | 2018 | Did not meet inclusion criteria or met exclusion criteria |
| Debs et al. | 2015 | Did not meet inclusion criteria or met exclusion criteria |
| Debs et al. | 2020 | Wrong study design |
| De la Cruz et al. | 2019 | Did not meet inclusion criteria or met exclusion criteria |
| Dijkhorst et al. | 2018-1 | Did not meet inclusion criteria or met exclusion criteria |
| Dijkhorst et al. | 2018-2 | Risk of redundant data report |
| Di Stefano et al. | 2019 | No article could be retrieved |
| D’Urso et al. | 2021 | Wrong study design |
| Elabd et al. | 2019 | No article could be retrieved |
| El Chaar et al. | 2017 | Did not meet inclusion criteria or met exclusion criteria |
| Elmaleh Mostafa et al. | 2019 | No article could be retrieved |
| Elsaher et al. | 2019 | Did not meet inclusion criteria or met exclusion criteria |
| Elzaidi et al. | 2019 | No article could be retrieved |
| Felsenreich et al. | 2016 | Did not meet inclusion criteria or met exclusion criteria |
| Felsenreich et al. | 2018 | Wrong study design |
| Felsenreich et al. | 2021 | Did not meet inclusion criteria or met exclusion criteria |
| Ferre et al. | 2017 | Did not meet inclusion criteria or met exclusion criteria |
| Ferro Echevarría et al. | 2019 | No article could be retrieved |
| Filip et al. | 2019 | Wrong study design |
| Flahou et al. | 2017-1 | No article could be retrieved |
| Flahou et al. | 2017-2 | No article could be retrieved |
| Flahou et al. | 2018 | Did not meet inclusion criteria or met exclusion criteria |
| Frieder et al. | 2020 | Wrong study design |
| Gadiot et al. | 2013 | Did not meet inclusion criteria or met exclusion criteria |
| Gerges et al. | 2021 | Wrong study design |
| Giorgi et al. | 2019 | No article could be retrieved |
| Guzmán et al. | 2017 | Did not meet inclusion criteria or met exclusion criteria |
| Haddad et al. | 2017 | Did not meet inclusion criteria or met exclusion criteria |
| Hernandez et al. | 2015 | Did not meet inclusion criteria or met exclusion criteria |
| Homan et al. | 2014-1 | Did not meet inclusion criteria or met exclusion criteria |
| Homan et al. | 2014-2 | Did not meet inclusion criteria or met exclusion criteria |
| Homan et al. | 2014-3 | Did not meet inclusion criteria or met exclusion criteria |
| Humes et al. | 2019 | Did not meet inclusion criteria or met exclusion criteria |
| Iannelli et al. | 2011 | Did not meet inclusion criteria or met exclusion criteria |
| Iannelli et al. | 2016 | Wrong study design |
| Ibarzabal et al. | 2018 | Did not meet inclusion criteria or met exclusion criteria |
| Ibarzabal et al. | 2019 | No article could be retrieved |
| Jamal et al. | 2020 | Wrong study design |
| Kraljević et al. | 2016 | Did not meet inclusion criteria or met exclusion criteria |
| Kraljević et al. | 2017 | No article could be retrieved |
| Kraljević et al. | 2021 | Wrong intervention |
| Kroll et al. | 2017 | No article could be retrieved |
| Landreneau et al. | 2018 | Did not meet inclusion criteria or met exclusion criteria |
| Liagre et al. | 2021 | Wrong study design |
| Marti-Fernandez et al. | 2020 | Did not meet inclusion criteria or met exclusion criteria |
| Maselli et al. | 2021 | Wrong intervention |
| Moon et al. | 2019 | Wrong outcome reported |
| Morais et al. | 2016 | Did not meet inclusion criteria or met exclusion criteria |
| Mora Oliver et al. | 2019 | Wrong outcome reported |
| Moszkowicz et al. | 2013 | Wrong study design |
| Moustafiz et al. | 2021 | No article could be retrieved |
| Musella et al. | 2019 | Wrong outcome reported |
| Nedelcu et al. | 2014 | Risk of redundant data report |
| Nedelcu et al. | 2015 | Risk of redundant data report |
| Neuberg et al. | 2018 | Did not meet inclusion criteria or met exclusion criteria |
| Nevo et al. | 2018 | Wrong study design |
| Noel et al. | 2014 | Risk of redundant data report |
| Noel et al. | 2020 | Wrong study design |
| Omarov et al. | 2020 | Wrong outcome reported |
| Osorio et al. | 2021 | Did not meet inclusion criteria or met exclusion criteria |
| Panomarenco et al. | 2014 | Did not meet inclusion criteria or met exclusion criteria |
| Park et al. | 2014 | Did not meet inclusion criteria or met exclusion criteria |
| Parmar et al. | 2017 | Did not meet inclusion criteria or met exclusion criteria |
| Pizza et al. | 2021 | Wrong study design |
| Poghosyan et al. | 2016 | Wrong study design |
| Poghosyan et al. | 2019 | Wrong study design |
| Poublon et al. | 2020 | Wrong outcome reported |
| Quezada et al. | 2016 | Wrong study design |
| Qui et al. | 2018 | Did not meet inclusion criteria or met exclusion criteria |
| Recarte et al. | 2016 | No article could be retrieved |
| Sabry et al. | 2021 | Did not meet inclusion criteria or met exclusion criteria |
| Sánchez-Pernaute et al. | 2020 | Wrong study design |
| Shimizu et al. | 2012 | Did not meet inclusion criteria or met exclusion criteria |
| Stefanidis et al. | 2013 | Wrong study design |
| Surve et al. | 2017 | Did not meet inclusion criteria or met exclusion criteria |
| van Wezenbeek et al. | 2015 | Did not meet inclusion criteria or met exclusion criteria |
| van Wezenbeek et al. | 2017 | Wrong outcome reported |
| Yan et al. | 2018 | Did not meet inclusion criteria or met exclusion criteria |
| Yilmaz et al. | 2017 | Wrong outcome reported |
| Yorke et al. | 2017 | Did not meet inclusion criteria or met exclusion criteria |
| Zaveri et al. | 2018 | Did not meet inclusion criteria or met exclusion criteria |
| Zaveri et al. | 2019 | Wrong study design |
