# Supplementary material for: Weight-Related Outcomes After Revisional Bariatric Surgery in Patients with Non-response After Sleeve Gastrectomy—a Systematic Review
Source: Obes Surg. 2023 May 20;33(7):2210–8. doi: 10.1007/s11695-023-06630-2 (PMC10289909; doi:10.1007/s11695-023-06630-2)
Supplement: Supplementary file 7 — (DOCX 14 kb) [file 11695_2023_6630_MOESM6_ESM.docx]

Supplementary Table 5: Detailed descriptions of inclusion criteria from all included studies

| Author | Definition of inclusion |
| --- | --- |
| Al Sabah et al. | N/A |
| Andalib et al. | “…defined a *failed* prior SG when any of the following was present at least 1 year after SG: (1) < 50% excess weight loss (EWL); (2) ≥ 20% weight regain of the weight lost” |
| Antonopoulos et al. | “Revisional surgery was proposed in patients with inadequate weight loss EWL≤ 50% or weight regain after SG” |
| Bashah et al. | “…revisional procedure for weight recidivism post-LSG.” |
| Chiappetta et al. | “Indications for conversion in the case of insufficient weight loss or weight regain included significant weight regain (> 15%), based upon whether they had gained > 15% of their 1-year postoperative weight, insufficient weight loss with an EWL < 50%” |
| Dapri et al. | N/A |
| De la Cruz et al. | “…primary indication for RBS was exhaustion of the restrictive component with stagnation of weight loss after LSG and a BMI of at least 35 kg/m2 or weight regain” |
| Dijkhorst et al. | “…BMI >35kg/m2, and all other criteria described in the European guidelines for bariatric surgery” |
| Homan et al. | “Progressive weight regain was defined as > 25% EWL regain with respect to the minimal weight after LSG, or when a patient met the criteria for bariatric surgery again, established by the International Federation for the Surgery of Obesity. Insufficient weight loss was defined as < 50% EWL without weight regain, or a when the International Federation for the Surgery of Obesity criteria for bariatric surgery were still met.” |
| Kraljević et al. | “Weight loss failure was defined as a %EWL< 50% or a body mass index (BMI) > 35 kg/m2 with persistence or recurrence of comorbidities” |
| Rayman et al. | “IWL was defined as failure to achieve a 50%excess weight loss (%EWL) at nadir weight following primary LSG, and WR was defined as achieving an initial 50% EWL but eventually regaining weight.” |
| Shimon et al. | “Indication for revision was failure of weight loss (insufficient weight loss (IWL)) or weight regain reflected by a body mass index (BMI) > 35 kg/m2…” |
