# Supplementary material for: Weight-Related Outcomes After Revisional Bariatric Surgery in Patients with Non-response After Sleeve Gastrectomy—a Systematic Review
Source: Obes Surg. 2023 May 20;33(7):2210–8. doi: 10.1007/s11695-023-06630-2 (PMC10289909; doi:10.1007/s11695-023-06630-2)
Supplement: Supplementary file 8 — (DOCX 14 kb) [file 11695_2023_6630_MOESM7_ESM.docx]

Supplementary Table 6: Definition of outcomes

| Author | Definition of outcomes |
| --- | --- |
| Al Sabah et al. | “Weight loss was expressed in terms of evolution of BMI and percent excess weight loss (EWL)” |
| Andalib et al. | “…weight loss after revision and described as both absolute and percent total weight loss (TWL), change in BMI, and percent EWL.” |
| Antonopoulos et al. | “…weight loss after revisional surgery (BMI, EWL)…” |
| Bashah et al. | “EWL% and TWL% were calculated with the weight before SADI or OAGB-MGB as a baseline.” |
| Chiappetta et al. | “Data collected included the following: … body mass index (BMI), EWL, total weight loss (TWL)…” |
| Dapri et al. | “…co-primary endpoint was the %EWL, calculated from the initial weight before ISG, and the ideal weight set at a BMI of 22 kg/m2…” |
| De la Cruz et al. | “…weight loss after RBS, expressed in percentage of total weight loss (%TWL)” |
| Dijkhorst et al. | “…percentage total body weight loss (%TWL, weight loss in kilograms at a follow-up time point divided by weight in kilograms measured before revisional surgery)” |
| Homan et al. | “%EWL (defined as weight loss divided by excess weight; the excess weight is defined as preoperative weight minus ideal weight, with the calculation of ideal weight as that equivalent to a BMI of 25 kg/m2) or percentage total weight loss (%TBWL; defined as weight loss divided by preoperative weight).” |
| Kraljević et al. | “Weight outcomes were recorded as follows: mean initial BMI, change in ΔBMI (initial BMI – postoperative BMI), %TWL defined as ((Initial Weight) – (Postoperative Weight)]/(Initial Weight) × 100) and %EWL defined as ( ( Initial Weight) – (Postoperative Weight)]/[(Initial Weight) – (Ideal Weight)). The weight corresponding to a BMI of 25 kg/m2 was considered as ideal.” |
| Rayman et al. | “Overall weight loss was expressed as percent total weight loss (%TWL) and measured between 4 timeframes: from initial weight to post-LSG nadir, initial weight to pre-revision weight, and initial weight to current weight. %TWL was also measured from pre-revision to post-revision nadir weight” |
| Shimon et al. | N/A |
