# Supplementary material for: Weight-Related Outcomes After Revisional Bariatric Surgery in Patients with Non-response After Sleeve Gastrectomy—a Systematic Review
Source: Obes Surg. 2023 May 20;33(7):2210–8. doi: 10.1007/s11695-023-06630-2 (PMC10289909; doi:10.1007/s11695-023-06630-2)
Supplement: Supplementary file 9 — (DOCX 14 kb) [file 11695_2023_6630_MOESM8_ESM.docx]

Supplementary Table 7: Follow-up rates grouped by type of revisional procedure

| Type of revisional procedure | Number of patients | Number of patients  1-year follow-up | Number of patients  2-year follow-up | Number of patients  3-year follow-up | Number of patients  4-year follow-up | Number of patients  5-year follow-up | % of patients  1-year follow-up | % of patients  2-year follow-up | % of patients  3-year follow-up | % of patients  4-year follow-up | % of patients  5-year follow-up |
| --- | --- | --- | --- | --- | --- | --- | --- | --- | --- | --- | --- |
| DS | 87 | 25 | 19 | 14 | 18 | 0 | 29% | 22% | 16% | 21% | 0% |
| SADI | 154 | 140 | 77 | 60 | 16 | 9 | 91% | 50% | 39% | 10% | 6% |
| GBP | 405 | 224 | 65 | 188 | 41 | 27 | 55% | 16% | 46% | 10% | 7% |
| Re-SG | 119 | 88 | 18 | 8 | 5 | 7 | 74% | 15% | 7% | 4% | 6% |
| OAGB | 281 | 125 | 174 | 21 | 0 | 0 | 44% | 62% | 7% | 0% | 0% |
| Total | 1046 | 602 | 353 | 291 | 80 | 43 | 58% | 34% | 28% | 8% | 4% |
