# Supplementary material for: Weight-Related Outcomes After Revisional Bariatric Surgery in Patients with Non-response After Sleeve Gastrectomy—a Systematic Review
Source: Obes Surg. 2023 May 20;33(7):2210–8. doi: 10.1007/s11695-023-06630-2 (PMC10289909; doi:10.1007/s11695-023-06630-2)
Supplement: Supplementary file 10 — (DOCX 46 kb) [file 11695_2023_6630_MOESM9_ESM.docx]

Supplementary Table 8: Technical details of SG and the different revisional procedures

**Characteristics of the sleeve gastrectomy**

Technical details of the SG were described in 4/12 studies (9, 17, 18, 22). The bougie size varied between 32 and 36Fr, the distance from the pylorus was reported ranging from 3 to 6 cm. Andalib et al. utilized a bougie with a size of 40 and 60Fr.

**Characteristics of the revisional procedures:**

*Gastric bypass*

Apart from Rayman et al. (24), the technical details of the conversional procedure from SG to GBP were presented by all authors. The length of the alimentary limb was at least 100 cm. Most surgeons stated a length of 150 cm for the alimentary limb (17, 19, 25) and between 50 and 70 cm for the biliopancreatic limb (9, 18, 19, 21, 25). Only Dijkhorst et al. (16) described a GBP configuration with a biliopancreatic limb exceeding the length of the alimentary limb. Three different types of GBP were detailed in the publication of Kraljević et al.: proximal GBP, type 2 distal GBP, and long BPL-GBP. The latter was applied in patients with insufficient weight loss or weight regain. Hence, this variation with a BPL-length of 100-150 cm and a common channel length of 100 cm was included in this review (15). Homan et al. modified their GBP technique under the study period: “Until 2009, patients with BMI < 50 kg/m2 had a short alimentary limb (SAL) of 100 cm (SAL; range 75–125 cm) and patients with BMI > 50 kg/m2 a long alimentary limb (LAL) of 150 cm (range 125–175 cm) using the same technique. Since then, all patients have received a LAL RYGB” (9).

*One-anastomosis gastric bypass*

Technical details of the conversion from SG to OAGB were propounded in 4/5 studies (15, 20, 21, 23). In all publications, the length of the biliopancreatic limb was 150-200 cm. A simultaneous re-SG or “trimming of the previous gastric sleeve” was routinely performed by Bashah et al. (20). Dilatation of the sleeve was regarded as indication for a re-SG in one study (21).

*Biliopancreatic diversion with duodenal switch*

Dapri et al., Andalib et al. and Homan et al. performed routinely a BPD/DS-procedure after previous SG with 100 cm common channel and 150 cm alimentary limb (9, 18, 22). The length of the common channel ranged from 60-100 cm and the alimentary limb from 150-190 cm in Shimon’s publication. A re-SG was added in three patients “when the sleeve was discovered as being excessively dilated” (25).

*Single-anastomosis duodeno-ileal bypass*

In 2/4 studies, the length of the alimentary limb was 250-300 cm (16, 20). De la Cruz et al. described a length of 250 cm and performed a simultaneous re-SG in 10/42 patients “based on the preoperative endoscopic (gastroscopic inversion possible) and intraoperative aspects (obvious large sleeve volume)” (23). An alimentary limb length of 0 cm was routinely chosen in Andalib et al.´s publication (18).

*Re-sleeve gastrectomy*

Surgical details of re-SG were depicted in all 4 studies. The bougie-size was varying between 34 Ch and 60 Ch (17-19, 22). During the timeframe of inclusion, Antonopoulos et al. introduced a staple line reinforcement (19). A routine leak-test was
